# Seeing the world through the eyes of a butterfly: visual ecology of the territorial males of *Pararge aegeria* (Lepidoptera: Nymphalidae)

**DOI:** 10.1007/s00359-021-01520-3

**Published:** 2021-10-28

**Authors:** Martin Bergman, Jochen Smolka, Dan-Eric Nilsson, Almut Kelber

**Affiliations:** Lund Vision Group, Department of Biology, Lund University, Sölvegatan 35, 223 62 Lund, Sweden

**Keywords:** Lepidoptera, Mate detection, Visual ecology, Spatial resolution, Visual environment

## Abstract

Combining studies of animal visual systems with exact imaging of their visual environment can get us a step closer to understand how animals see their “Umwelt”. Here, we have combined both methods to better understand how males of the speckled wood butterfly, *Pararge aegeria*, see the surroundings of their perches. These males are well known to sit and wait for a chance to mate with a passing females, in sunspot territories in European forests. We provide a detailed description of the males' body and head posture, viewing direction, visual field and spatial resolution, as well as the visual environment. *Pararge aegeria* has sexually dimorphic eyes, the smallest interommatidial angles of males are around 1°, those of females 1.5°. Perching males face the antisolar direction with their retinal region of the highest resolution pointing at an angle of about 45° above the horizon; thus, looking at a rather even and dark background in front of which they likely have the best chance to detect a sunlit female passing through the sunspot.

**Supplementary Information:**

The online version contains supplementary material available at 10.1007/s00359-021-01520-3.

## Introduction

Mating success strongly depends on the probability of encountering a suitable mate, and mate location strategies vary widely among animals (Thornhill and Alcock 1983). In butterflies, two main strategies have been described, patrolling and perching (Scott 1974; Rutowski 1991; Wiklund 2003). Patrolling males spend most of their day on the wing, flying and searching for females, while perching males sit and wait for receptive females, often defending a territory at the perching site. In some species, perching sites are in the vicinity of resources used by females, such as larval host plants (Baker 1972; Courtney and Parker 1985; Rosenberg and Enquist 1991; Lederhouse et al. 1992) or adult food sources (Suzuki 1976; Fischer and Fiedler 2001). However, in other species, males perch in and defend non-resource-based sites, which are often described by typical landscape structures such as gullies (Cordero and Soberon 1990), elevations and hilltops (Shields 1967; Lederhouse 1982; Alcock 1987), trees or bushes (Wickman 1985) or large sun spots on the forest floor (Davies 1978; Wickman and Wiklund 1983; Bergman and Wiklund 2009).

It can therefore be hypothesized that males choose these locations for perching because they increase their chances to mate with a female. This advantage could be due to several reasons. They could enhance the chance of encountering receptive females (Parker 1978; Rutowski 1984). An additional reason could be that passing females are more easily detected in these locations. Perching butterfly males detect females visually (Bergman and Wiklund 2009); hence, selection should favour males that establish perching sites where females are not only likely to be encountered, but also easily seen (Rutowski 1991). How easily a male can detect a female from his perching site, should strongly depend on the contrast between the female and the background. This dependency has only been investigated for one species of perching butterflies, *Asterocampa leilia* (Bergman et al. 2015). We suggest that to understand this dependency in many species, a first step should be an exact description of the visual scene that serves as background. Although the importance of the light environment in forest has been pointed out a long time ago (Endler 1993) methods allowing a detailed description have been scarce, the method described by Nilsson and Smolka (2021) being especially promising.

Detection of females in front of a background strongly depends on the visual abilities of the males, and more specifically, on the spatial resolution of their compound eyes. Spatial resolution can be estimated from the angle between the optical axes of two adjacent ommatidia, the interommatidial angle, in the frontal part of the visual field of view (Land 1997a; b; Rutowski and Warrant 2002; Rutowski et al. 2009). This angle depends on the curvature of the eye, and thus eye size, which scales with body size. Larger butterfly species have larger eyes and higher spatial resolution (Rutowski et al. 2009), and in many butterfly species, males have larger eyes than females, and; thus, higher spatial resolution than females, possibly allowing them to detect mates from a larger distance (Rutowski 2000a; Rutowski and Warrant 2002). Similar results have been found in other insects that visually detect mates, for instance flies (e.g. Collett and Land 1975; Zeil 1983), honeybees (Menzel et al. 1991) and carpenter bees (Somanathan et al. 2017).

In this study, we used a new combination of methods to see the environment through the eyes of male speckled wood butterflies, *Pararge aegeria*, from their perching sites. *P. aegeria* males perch and defend sunspot territories in forests in central and northern Europe (e.g. Davies 1978; Wickman and Wiklund 1983; Bergman and Wiklund 2009; Wiklund and Friberg 2011). Similar to other nymphalid butterflies, they have apposition compound eyes with three spectral types of photoreceptors sensitive to ultraviolet (UV, maximal sensitivity at 360 nm), blue (460 nm) and green (530 nm) light (Paul et al. 1986, and see van der Kooi et al 2021). Males have earlier been found to possess larger eyes than females (Rutowski 2000a) and are known to detect females visually. They also engage in territorial contests with and chase away intruding males (e.g. Davies 1978; Wickman and Wiklund 1983). Males prefer larger to smaller sunspots (Bergman and Wiklund 2009) and males perching in a large sunspot have a higher mating success than males perching in a small sunspot (Bergman et al. 2007). The exact mechanism for this mating success asymmetry is still unknown. Previous studies have shown that females do not have a preference to visit large sunspots when basking (Bergman et al. 2007). It has; therefore, been suggested that males are more likely to discover a passing female in a large sunspot, because large sunspots (1) enhance the visibility of females (Bergman et al. 2007; Bergman and Wiklund 2009) and (2) help males to reach an optimal body temperature for following females or intruders (Van de Velde et al. 2011).

Hence, it is still largely unknown what constitutes territory quality in this mating system and what maintains territorial behaviour. Here we pursue this question by studying the visual and behavioural ecology of perching males with the aim to better understand how males detect and choose their perch sites, and how they position themselves in a perch to optimize their chances to detect a female. We use a new combination of methods to analyse how the choice of perching site shapes the visual environment within which male *P. aegeria* detect their potential mates. To achieve this goal, we (1) established the visual field and eye maps of the interommatidial angles of the eyes of *P. aegeria*, (2) measured sun spot size and sun direction as well as posture and orientation of perching males and (3) used a new camera technique (Nilsson and Smolka 2021) to quantify the environmental light field of a perch location. These combined data will hopefully allow others to answer the next questions. We suggest that this combination of methods may help us to better understand choices of visual environments also in other species.

## Materials and methods

### Animals and field site

Behavioural observations were conducted in May–August 2015 in Kullaberg, Sweden. The site, known as Ransvik (56.2930 N, 12.4780 E), is located in southern Sweden approximately 100 km northwest of Malmö and has been used in previous studies of *P. aegeria* (Bergman and Wiklund 2009; Wiklund and Friberg 2011). The habitat consists of an open forest dominated by beech (*Fagus sylvatica*) and oak (*Quercus robur*), undergrowth dominated by *Rubus fruticosus* and *Lonicera periclymenum*, and a forest floor dominated by the grasses *Melica nutans*, *Dactylis glomerate* and *Brachypodium sylvaticum*. The *P. aegeria* population at Ransvik is well known and its phenology and dynamics have been studied for over 20 years (Wiklund and Friberg 2011).

### Visual fields and interommatidial angle maps

Three males and three females of *Pararge aegeria* were collected at the field site. We followed standard procedures to map interommatidial angles in the frontal part of the visual field of males and females of *P. aegeria* (Land and Eckert 1985; Rutowski et al. 2009; Kelber et al. 2011; Somanathan et al. 2017). In short, an immobilised butterfly was mounted at the centre of curvature of a Leitz goniometer with the flat posterior eye edge parallel to the plane of the goniometer stage, and placed beneath an optical apparatus consisting of a Canon MD150 digital video camcorder and an inverted Hasselblad Distagon 1:3.5 60 mm camera objective (with 80 mm back focal distance). This optical apparatus acted as a microscope that allowed single images to be captured from the Canon camcorder.

The butterfly’s head was positioned such that the goniometer axes were lined up with the dorsal–ventral (yaw), anterior–posterior (roll) and left–right (pitch) axes of the head and the back edge of the eyes aligned parallel to the stage. With the stage horizontal, the butterfly’s frontal visual field was oriented vertically upwards, looking into the rear lens of the Hasselblad objective. The eyes were illuminated by white light from a light emitting diode (LED), reflected into the observation path through a 45° half-silvered mirror just beneath the lens. This orthodromic illumination made a luminous pseudopupil (the facets looking into the lens) visible. After locating the pseudopupil and focusing the image, the LED was covered to allow for dark adaptation of the photoreceptors. As the bright (which is required to obtain a bright image of the luminous pseudopupil). Immediately after the LED was uncovered again, a photo was taken (see Supplementary Fig. S1 for an example photo).

We used the goniometer to tilt the butterfly’s head in defined angular steps of 10° in latitude and longitude, with latitude = 0° and longitude = 0° defined as the anterior orientation, and took a series of images of the pseudopupil. Barium sulphate powder was sprinkled lightly on the eye to provide landmarks. Due to the structure of the apparatus we could not go beyond latitudes of + 70° or − 70° or a longitude of 100°. Hence, our observations of the appearance and location of the pseudopupil were restricted to the frontal eye region, which is, in any event, the region of greatest interest for this study. In addition, at 10° intervals of latitude, the front edge of the visual field was determined as the longitude, at which the pseudopupil disappeared. For determination of the rear edge of the visual field, one male head was mounted in the opposite orientation (with the flat hind edge of the head pointing upwards in the goniometer).

Eye maps were generated by converting the position of the pseudo pupil from angular coordinates to eye coordinates (in the lattice of facet rows). From each image, we determined the coordinates (as the *x*- and *y*-rows defined in Supplementary Fig. 1) of the facet at the centre of the pseudopupil. Using established formulae that correct for latitude distortions in the projection (Land and Eckert 1985) in a custom-made program (Rutowski et al. 2009), we calculated the average local interommatidial angle Δ*φ* for each combination of latitude and longitude. These data were plotted on a sphere representing the three-dimensional space around the animal, and contours were interpolated to connect regions of space viewed by parts of the eye with the same Δ*φ*. Minimum interommatidial angles of males were used to filter images taken at the perch sites of males (see below).

### Behavioural observations and sunspots

Between 10 h and 17.30 h local time, we identified sunspots that were used as perching sites by male *P. aegeria* in the field. To decide whether the sunspot was used as a territorial site, we assessed the male’s willingness to stay in the sunspot and return to the same perch after a short flight. We did this by either waiting a few minutes until the perching male performed a spontaneous flight or encouraging the male to take off by tossing a small piece of bark into the sunspot, which invariably caused the male to take flight and chase the intruding object. If the male, after such a short flight, returned to the same sunspot, we considered that sunspot to be used as a territorial site by that male. Conversely, if the male did not return to the same sunspot, we considered that the sunspot was not used as a territorial site but merely as a place chosen for basking.

Once a territorial male was located, we described the size of the sunspot territory by measuring its length and width (sun spots rarely had square or circular shapes; the larger dimension was defined as length, the shorter as width) as well as the distance between the points where the male perched within the sunspot. We studied 27 individual perching males for two consecutive landings, and 16 of these for a third landing, before they abandoned the sunspot as a result of the continuous disturbance. Thus in total, we observed 70 individual perch positions by the 27 males. When possible, we recorded the type of substrate (rock or vegetation) the height at which the male perched. For a subset of 14 occupied sunspots (of at least 1 m × 2 m size, see the Supplement data file), we located the nearest sunspot not occupied by a male, with the goal to test which visual traits of a sunspot predict its quality as a territory.

### Body orientation and posture of perching males

For the first landing of each male, we recorded the compass direction that the male faced relative to north while perching (body azimuth) and whether he had his wings open or closed. We also determined sun azimuth and elevation at the time of observation.

**Fig. 1:**
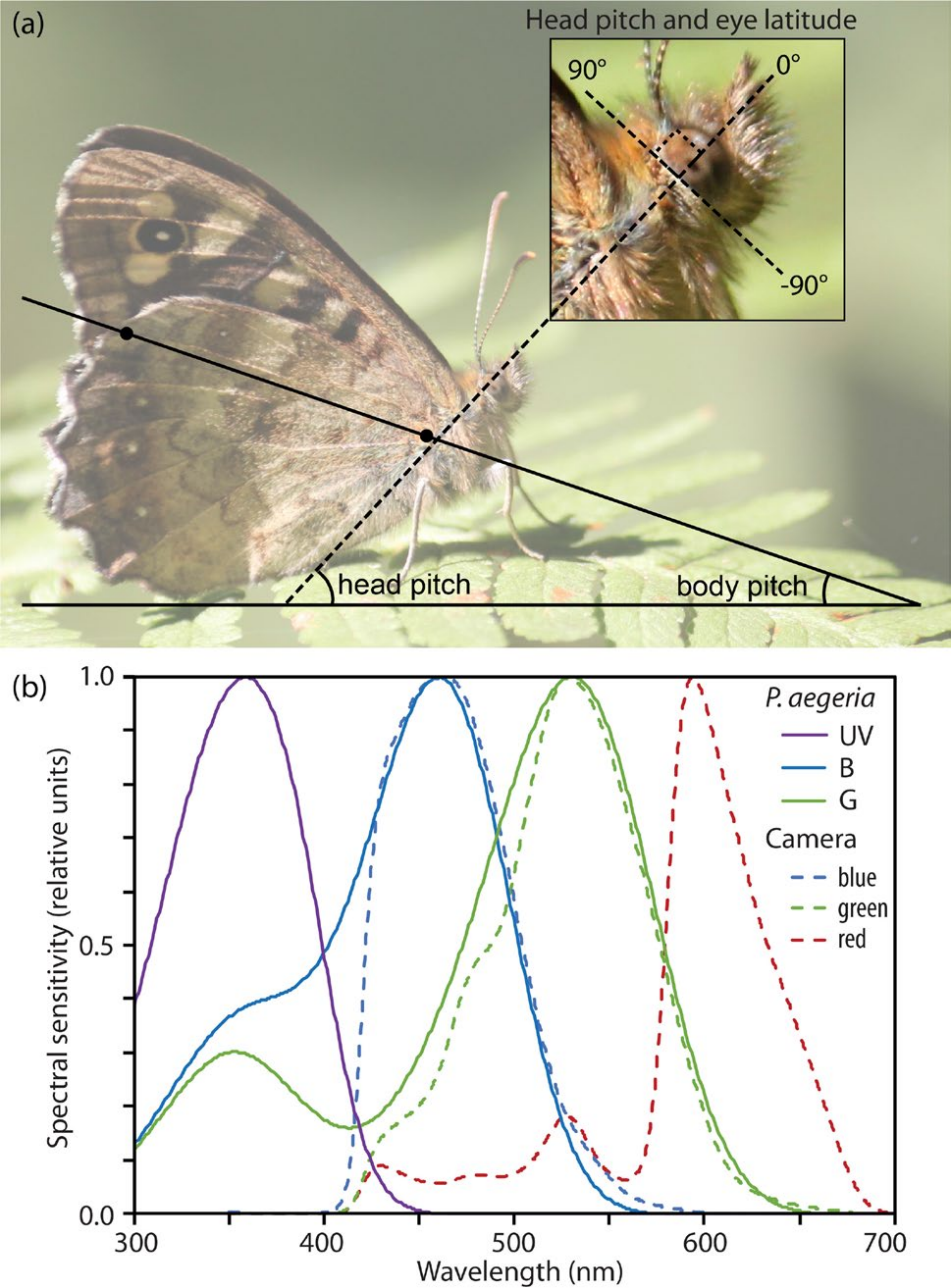
Methods. **a** Head and body pitch of a perching male, determined from photographs taken with a level camera. The inset shows the definition of eye latitude angles used for mapping interommatidial angles, with 0° is horizontal and 90° dorsal. Note that the eye horizon of the perching male points at about 45° elevation. **b** Spectral sensitivity of the photoreceptors on *P. aegeria* (solid lines, from left to right UV—ultraviolet-sensitive, B blue-sensitive and G green-sensitive photoreceptors) and the Nikon camera (dashed lines, from left to right blue, green and red channel) used to image the visual environment. Note the close similarity of the blue and green channels of butterfly and camera

To quantify the body posture we took photographs of the males after the first landing using a digital camera (Canon EOS 500D fitted with a Canon EF-S 55-250 mm *f*/4–5.6 IS Zoom Lens and an attached spirit level). Photographs were taken from the same height at which the male was perched, in the horizontal direction and at right angle from the body axis (Fig. 1a). We were able to take photos of 25 of the 27 observed males. From these photos, we assessed the head and body pitch of the males (see Supplementary Fig. S2 for example photographs).

The head pitch is the angle between the horizontal and the eye equator (0° elevation; inset in Fig. 1a). In the spherical eyes of the butterflies, the eye horizon is perpendicular to the straight back edge of the eye, which is parallel to the back edge of the head (± 90° elevation, inset in Fig. 1a). The body pitch describes the body posture with respect to the horizontal. However, in a *P. aegeria* male perching with closed wings, a large part of the body (the thorax and abdomen) is hidden behind the wing (Fig. 1a), making it impossible to assess the pitch of thorax and abdomen. Instead, we used wing orientation as an approximation of body pitch, by drawing a line between two points on the ventral side of the hind wing, the point where the wing vein *R*_s_ meets the outer margin of the wing and the point at the wing base where the hind wing attaches to the thorax (line and solid dots in Fig. 1a). The angle between this line and the horizontal was used as the body pitch of perching males. Assessing this angle was impossible in males sitting with open wings, leaving us with body pitch measurements from only 17 males. To analyse whether males adjusted body posture depending on substrate, we also determined the slope of the substrate, whenever possible (13 males).

### Imaging the visual environment of perch sites

To quantify the environmental light field that males choose with their perch site, we determined the light distribution around the animals from calibrated digital photographs. The photographs were taken using a digital camera (D800E or D810: Nikon Corp., Shinjuku, Japan) fitted with a fisheye lens (Sigma 8 mm F3.5 EX DG: Sigma Corp., Kawasaki, Japan). The camera was calibrated in order to correct for different exposure times, ISO speed settings and apertures, as well as for the effect of vignetting (i.e. lower exposure at the image edges) to provide absolute radiance measurements for each pixel (Nilsson and Smolka 2021). The camera has three spectral channels, sensitive in the blue (maximal sensitivity at 463 nm), green (528 nm) and red (593 nm) part of the spectrum (Fig. 1b; Nilsson and Smolka 2021). Although the eyes of *P. aegeria* have a UV-sensitive photoreceptor (maximal sensitivity at 360 nm, Paul et al. 1986) and lack a red-sensitive channel, the sensitivities of the butterfly’s and camera’s blue and green channels are remarkably similar (Fig. 1b). For each location and orientation, three differently exposed images were taken covering a total of 6EV (i.e. a 64-fold increase in exposure), making sure that no part of the image was overexposed in the darkest of the images. By combining these images into a High Dynamic Range (HDR) representation, we can increase the dynamic range of the camera to cover the full dynamic range of natural scenes. The camera was levelled using a hot-shoe mounted spirit level. To quantify light intensity information, we then averaged spectral photon radiance along the horizontal dimension to obtain a vertical profile of intensities (described by their median, inter-quartile range and 95% range) for each of the three colour channels and for an average “white” channel. We assume that the green receptor channel of the butterflies is used as main luminance channel, as is the case in other insects (e.g. Srinivasan and Lehrer 1988).

For each male, four sets of photographs were taken: (1) from the exact point where the male first perched, one photo was taken towards the azimuth direction he was facing. (2) In the same position, a second photo was taken towards the opposite direction (180°). (3) In the unoccupied sunspot (14 sunspots, see above) one photo was taken in the direction the male was facing (in the corresponding occupied sunspot) and (4) the last photo in the opposite direction (180°).

Calibrated images were filtered in custom-made software, written in Matlab 2017a (MathWorks, Natick, USA), to approximate the spatial resolution of the butterfly eyes. Since the acceptance functions of photoreceptors of the *P. aegeria* eye are not known, the minimal interommatidial angle determined from the eye maps (see above) was used as an estimate of maximum potential acuity. Information theory predicts that with interommatidial angles of 1° ideal sampling would be achieved with an acceptance angle of 2° (Land 1997a, b), but many insects have narrower acceptance functions in parts of their eye, among these other Nymphalid butterflies, in which both angles are similar (Frederiksen and Warrant 2008). We applied two Gaussian filters with a half width (full width at half maximum; FWHM) of 1° and 10°, in order to get an estimate of the information available to the animals in the high-frequency and low-frequency spatial domain. Filter kernels were accurately calculated to take into account the distortion of the photographs by the camera lens. We then calculated the median radiance that would be available to an animal from these filtered images (assuming neuronal spatial sampling that matches the filter width). Radiance contrast C_R_ between two neighbouring receptive units calculated as unsigned Michelson’s contrast *C*_R_ =|*I*_1_ − *I*_2_|/(*I*_1_ + *I*_2_), where *I*_1_ and *I*_2_ are the radiant intensities measured by the two receptors. It is generally assumed that high-resolution spatial vision, and specifically the visual channels used for detecting moving stimuli, use a colour-blind green receptor signal; thus, we did not include colour contrast in the analysis.

## Results

### Visual field and interommatidial angles of *P. aegeria*

**Fig. 2:**
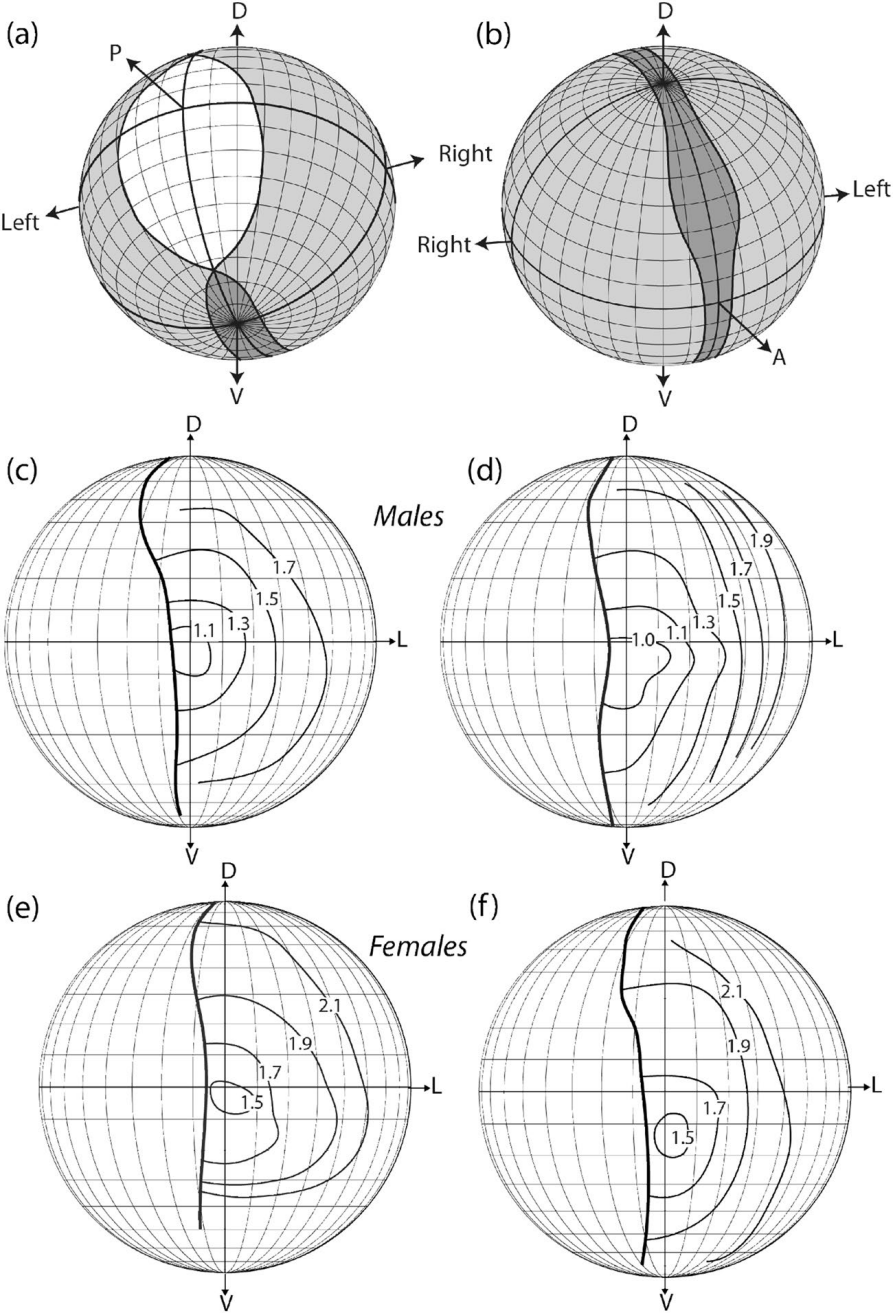
Visual fields and interommatidial angles. **a**, **b** Visual fields of a male *P. aegeria*. light grey: monocular visual field; dark grey: binocular visual field; white: blind angle. **c**, **f** Isolines indicating interommatidial angles in the frontal visual field of the eyes of two male (**c**, **d**) and two female (**e**, **f**) *P. aegeria*. *A* anterior, *D* dorsal, *L* lateral, *P* posterior

Male *Pararge aegeria* have almost panoramic vision, excluding only a narrow angle backwards where the view would anyway be blocked by the animal’s own thorax, abdomen and wings (Fig. 2a, b). We found a distinct sexual dimorphism in the interommatidial angles. Males have interommatidial angles smaller than 1.5° in large parts of their frontal visual field, down to ≈1°, in a small fronto-ventral area at an elevation between + 0 and − 10° on the eye; thus, around and slightly below the eye equator (Fig. 2c, d). In females, the smallest interommatidial angles, found in the same eye region, were in the range of 1.5° (Fig. 2e, f).

### Behaviour

The occupied sunspots had a median length of 8 m and a median width of 4 m but varied considerably (50% of lengths were between 5 and 10 m, 50% of widths between 3 and 5 m; *n* = 20, for details see Supplement to Bergman Pararge aegeria.xlsx). When a male alighted in the sunspot after a territorial scouting flight he most often perched on vegetation such as a leaf or a branch of the surrounding trees, bushes or grasses. 91% of all 70 observed perch positions by 27 males were on vegetation. Of the 63 perches for which height above ground level was measured, only 4 (6%) were directly on the ground, on some rock or stone on the forest floor. Males perched at a median height of 35 cm, and 50% of perches were within 15 and 50 cm above ground. There was no significant difference in perch height between the first, the second and the third landing that a male did during the observations (Repeated Measures ANOVA: *F*_2,30_ = 0.26; *p* = 0.77). The first and the second perch had a median ground distance of 75 cm (with 50% of the distances between 10 and 95 cm, *n* = 14) and so did the second and third perch (50% of distances were between 5 and 122 cm, *n* = 12). In summary, after a territorial scouting flight, a male usually alighted less than 1 m from the previous perch position. Given the size of the occupied sunspots, each male thus used a relatively small area for perching.

### Body posture: azimuth and pitch

**Fig. 3:**
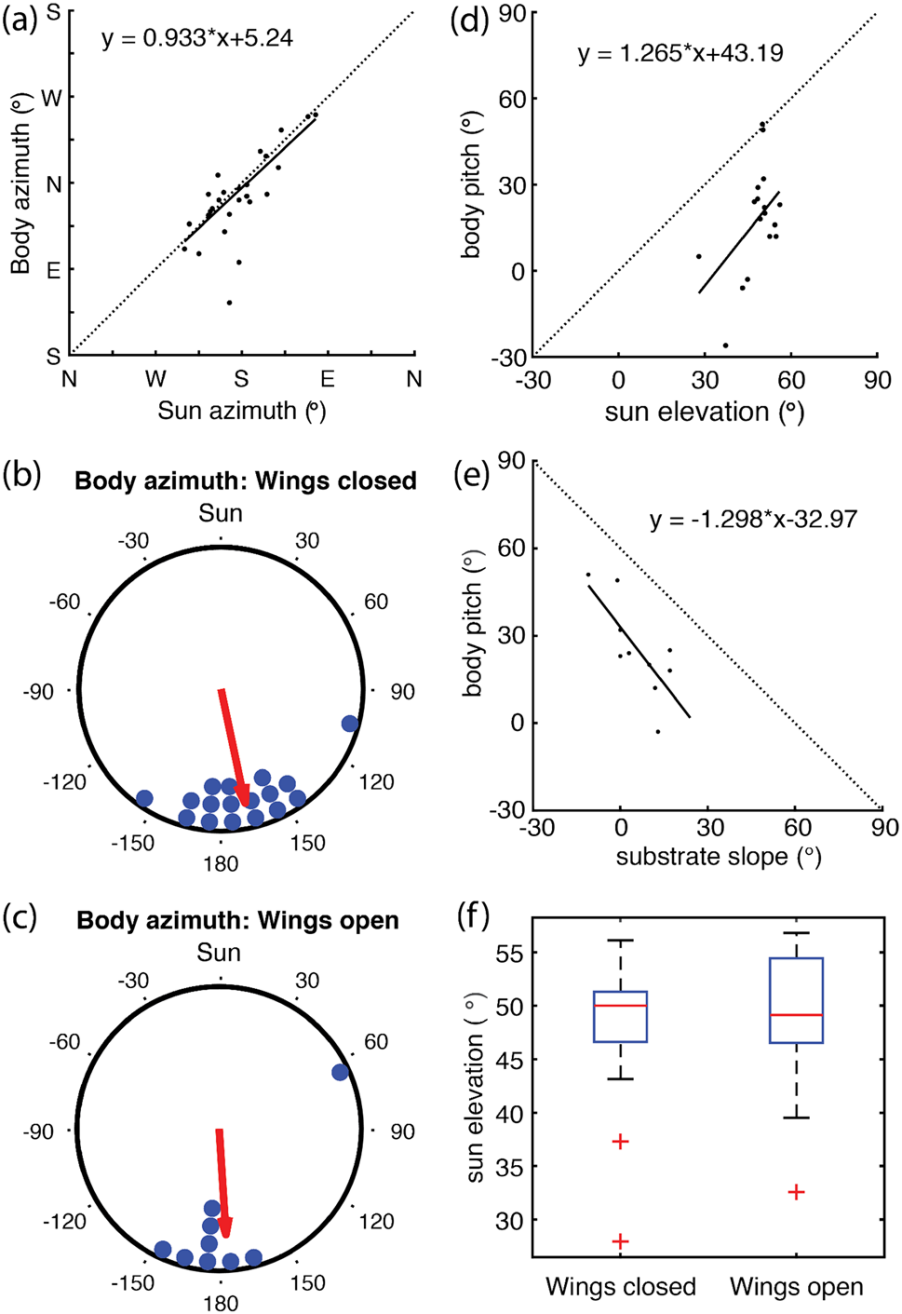
Body azimuth, body pitch and wing posture as function of sun position. **a** Body azimuth is closely correlated with sun azimuth: the male looks into the anti-solar direction [Pearson’s rho: 0.74 (*p* < 0.001), circular rho: 0.77 (*p* = 0.003)]. **b**, **c** This is independent of wing posture. Body pitch depends on (d) solar elevation (Pearson’s rho: 0.51, *p* = 0.036; circular rho: 0.51, *p* = 0.058) and **e** substrate slope (Pearson’s rho: − 0.76, *p* = 0.011; circular rho: − 0.76, *p* = 0.053). Dotted lines in **d** and **e** indicate the expected 45° angle and are not meant to indicate expected relationships themselves. Within the observed range, wing posture does not depend on solar elevation (Wilcoxon rank sum test: *Z* = 0.13, *p* = 0.90)

The azimuth of the body axis and; thus, the direction which males faced while perching, was non-randomly distributed (Rayleigh test: *Z* = 14.3; *p* < 0.001; concentration *κ* = 2.30), especially when measured relative to the sun’s azimuth (Rayleigh test: *Z* = 20.1; *p* < 0.001; concentration *κ* = 4.48). In fact, the animals consistently oriented in a way that put the sun at their backs (Pearson’s correlation, *ρ* = 0.74, *p* < 0.001; for details see Supplement to Bergman Pararge aegeria.xlsx). Whether males perched with open (*n* = 8) or closed (*n* = 17) wings did not depend on sun elevation (Fig. 3f), and there was no difference in body azimuth relative to the sun between males of these two groups (Fig. 3b, c; Wilcoxon rank-sum test: *Z* = 1.11, *p* = 0.27). Body azimuth was not affected by the perch height of the male either (circular–linear correlation: *r* = 0.14; *p* = 0.65).

**Fig. 4:**
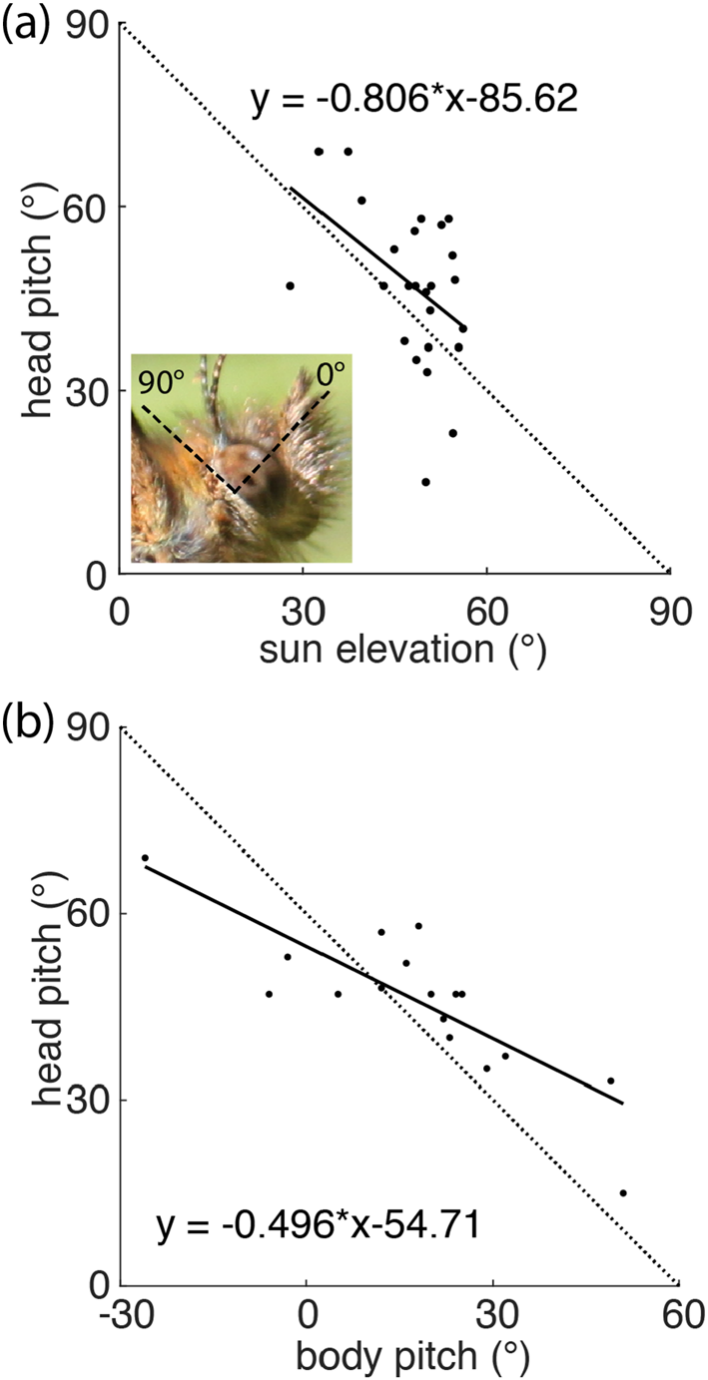
Head pitch depends on solar elevation. **a** Head pitch depends on solar elevation such that the butterfly keeps the sun at 90° (dorsally) in the visual field, see inset. *n*, Pearson’s rho: − 0.44, *p* = 0.027; circular rho: − 0.44, *p* = 0.048. **b** Head pitch also depends body pitch (*n* = 17, Pearson’s rho: − 0.83, *p* < 0.001; circular rho: − 0.83, *p* = 0.044)

The body pitch angle (Fig. 1) of perching *P. aegeria* males depended both on the sun elevation (Fig. 3d) and on the slope of the substrate (Fig. 3e). Males perched with their head pitch axis tilted upwards, on average 47° (± 13°, standard deviation) above the horizon (Fig. 4a, b). Head pitch was inversely correlated with sun elevation, which means that males kept the sun at 90° elevation in the visual field of their eyes, in an eye region with low spatial resolution (large interommatidial angles). In contrast, the eye region with the highest spatial resolution (i.e. smallest interommatidial angles), around and slightly below the eye equator, was facing the background at roughly 30°–60° above the horizon. Head pitch (Fig. 1) was inversely correlated with the body pitch of the male (Fig. 4b), indicating that males actively controlled head pitch to be constant and independent of body pitch and substrate angle.

### The visual environment as seen from the perch

**Fig. 5:**
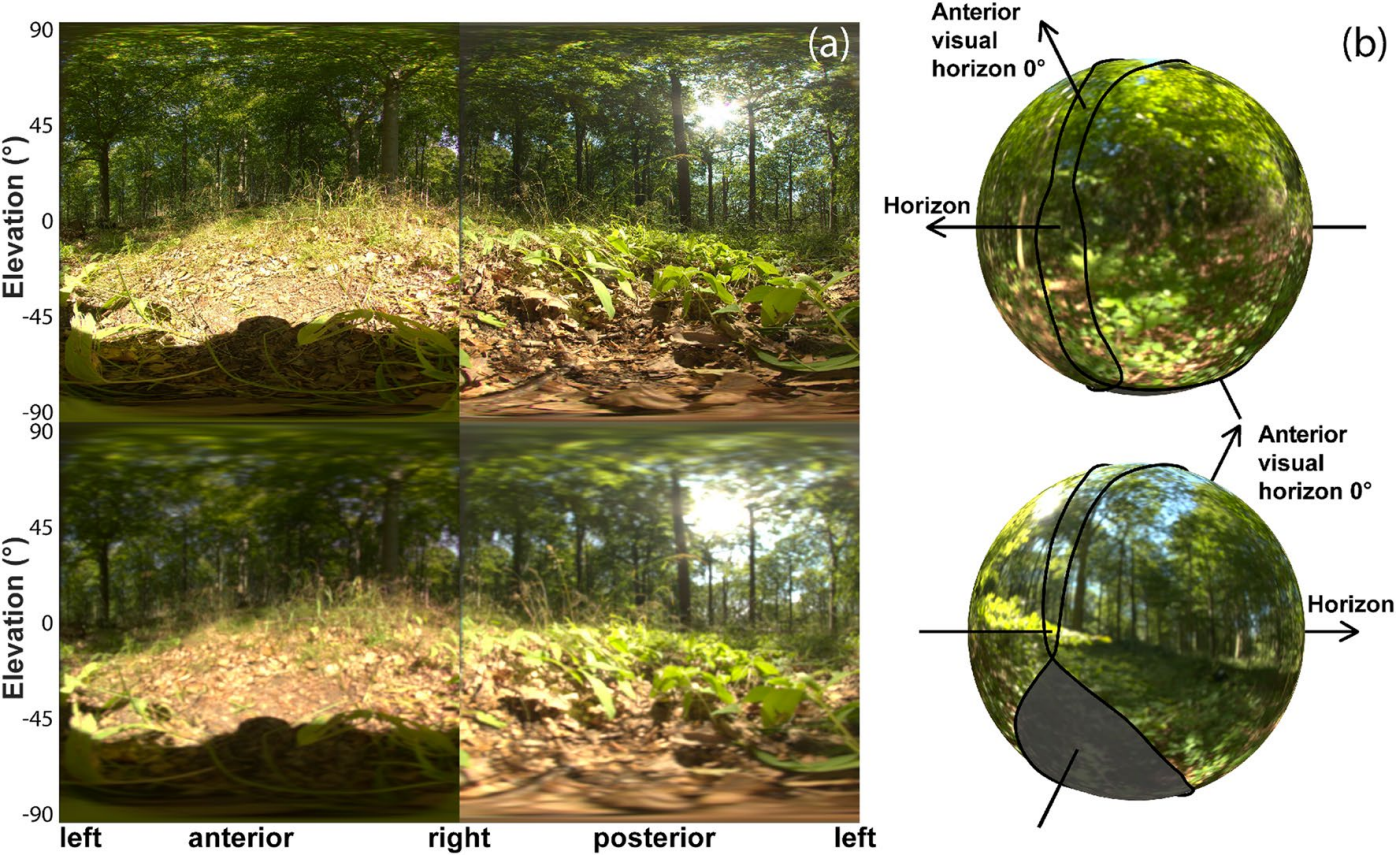
**a** Example for an anterior (left) and posterior (right) scene as seen by a male from his perch. Upper row shows the unwarped hemispheric images, lower row shows the same images filtered with 1° resolution, the highest resolution of male *P. aegeria*. Note that the dark shadow of the photographer in the anterior images is an artefact not present in the natural situation. **b** The panoramic views are projected onto the visual field of *P. aegeria* male, indicating that the male looks at the rather uniform green leaf cover with its eye region of highest resolution around the eye horizon

Figure 5a presents examples of hemispherical images remapped to an equirectangular projection (upper row) taken from the perch of a male into the azimuth of his viewing direction (anterior image, left) and in the opposite direction (posterior image, right), and the filtered versions of the same images, for 1° maximal resolution of the male eye (lower row). As the male was facing away from the sun, the sun appears with high contrast in the posterior (right) image, while the shadows of photographer, camera and tripod are darkening the lower middle part of the anterior (left) image. The projection of the visual environment onto the sphere representing the visual field of a male *P. aegeria* (Fig. 5b) indicates which parts of the scene are seen in the binocular and the monocular fields of view, and that only a small part of the substrate is invisible to the males, in the blind angle.

**Fig. 6:**
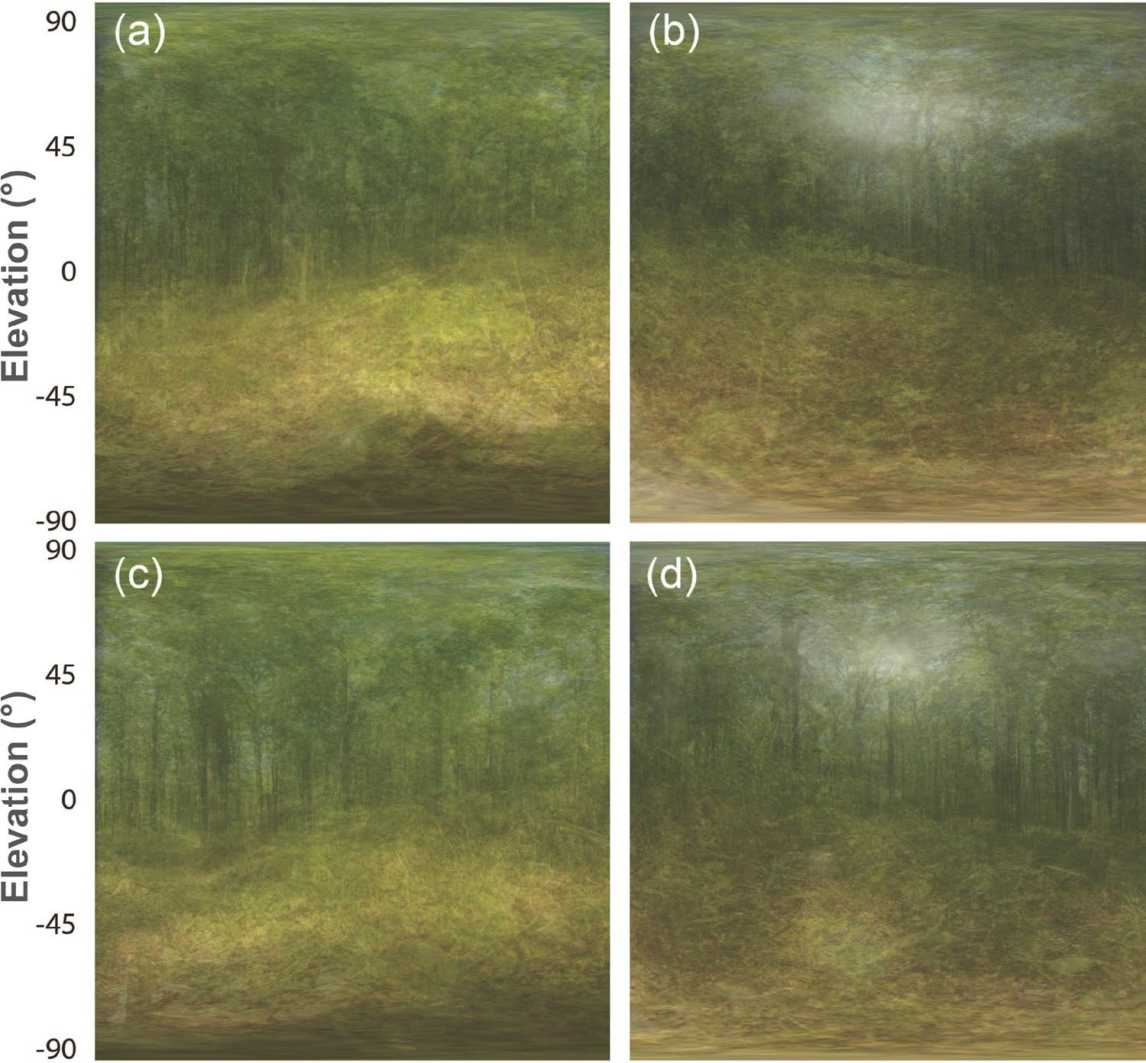
Mean anterior (left) and posterior (right) scenes averaged from 27 pictures taken in occupied (**a**, **b**) and 14 unoccupied (**c**, **d**) sunspots. Again, note that the dark shadow in the lowest elevation in the anterior scenes is caused by the photographer’s presence

The averages of all images taken at 27 occupied sunspots (Fig. 6a, b) show the same general pattern. This is similar even for unoccupied sunspots, which, however, look somewhat darker (*n* = 14; Fig. 6c, d).

**Fig. 7:**
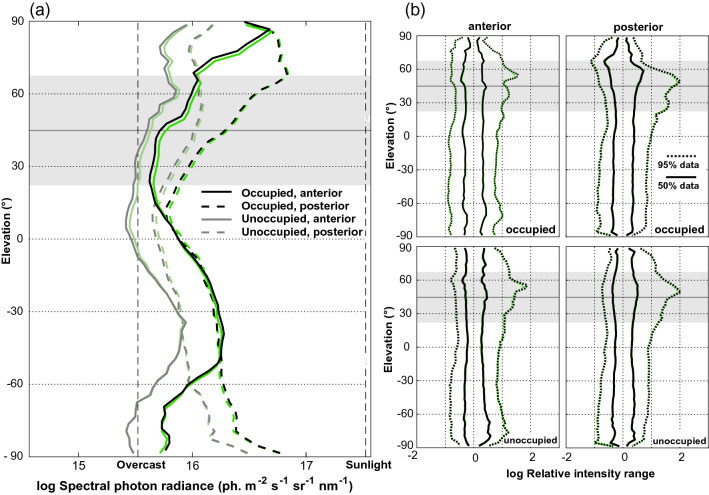
**a** Median vertical radiance profile in the anterior and posterior scenes in occupied and unoccupied sunspots. Black/grey lines show the overall radiance in all spectral channels, green/light green lines the profile in the green channel. In the range between 30° and 60° elevation, the anterior scenes (solid lines) show lower and more even profiles than in the posterior scenes. **b** Intensity range profile in typical scenes. The anterior scenes of occupied sunspots show a smaller intensity range, indicating a more even background in front of which passing females can more easily be spotted. The shaded area indicates the region, which the males views with highest resolution

**Fig. 8:**
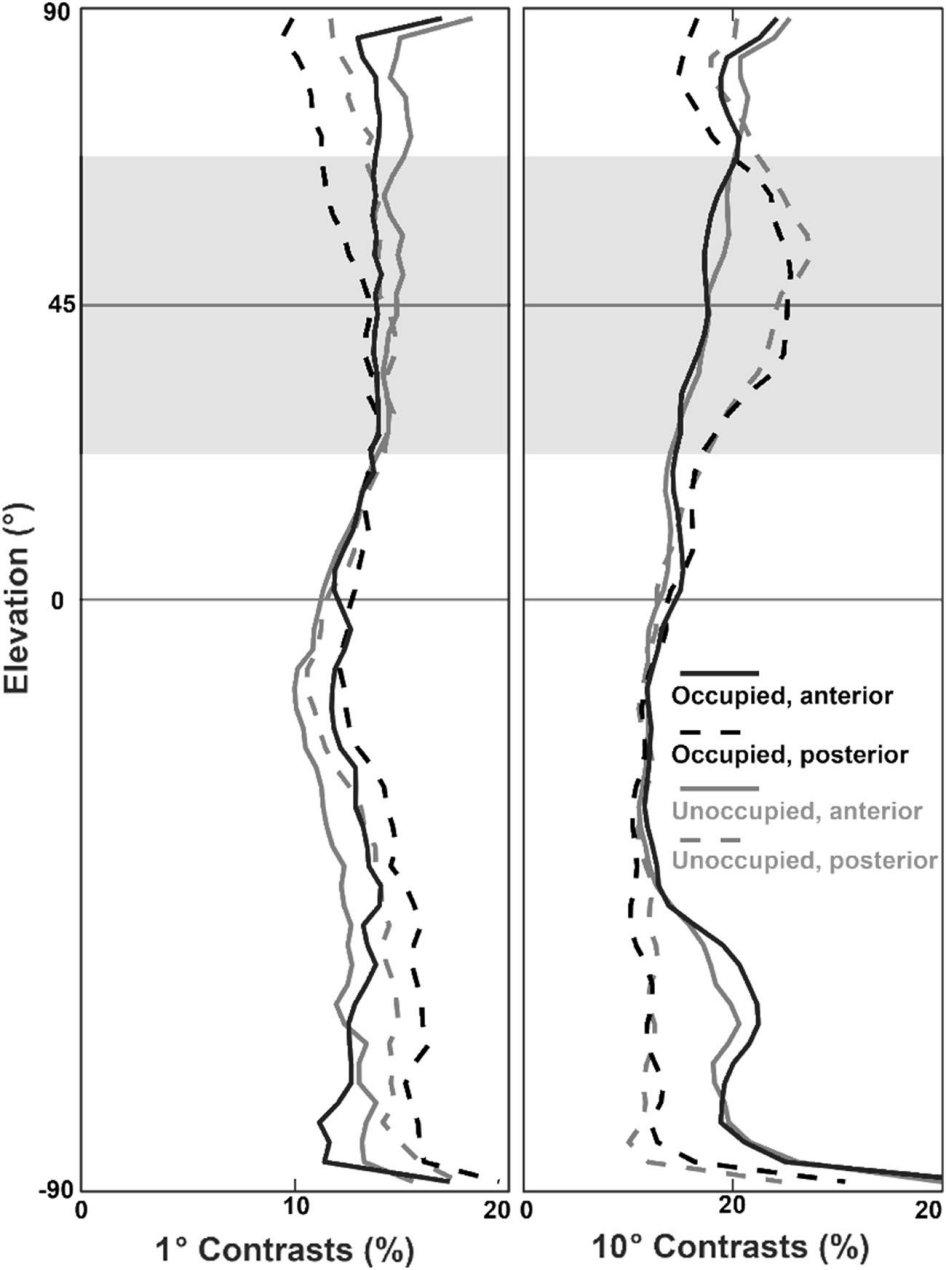
Median contrast profiles in the anterior and posterior scenes in occupied and unoccupied sunspots. In both spatial ranges, anterior scenes show more even profiles, and in the 10° range also much lower contrast values, allowing for better detection of passing females, specifically in the range into which males look with their visual field of highest spatial resolution (grey shading)

The image analysis confirms that occupied sunspots are brighter than unoccupied sunspots, both in the anterior and the posterior visual field (Fig. 7). If we focus on the region into which males look with the eye region of the highest spatial resolution, anterior and at an elevation of between 30° and 60° above the horizon (grey shaded zone in Fig. 7a), the intensities are relatively low, and stay within a narrow range of less than 1 log unit (Fig. 7b). In this elevation, the contrasts in the image (see “Methods” for definition) were also relatively low and rather constant (Fig. 8), both when assuming 10° and 1° spatial range indicating a uniform background. In the posterior part of the visual field, in which the butterflies see the sun, intensities as well as contrasts appear to be higher, specifically in the 10° spatial range.

## Discussion

We have combined classical measurements and a new imaging format to describe the visual environment of perching males of *Pararge aegeria*. Males have the highest spatial resolution (1°) frontally around their eye horizon, which they pitch to around 45° elevation, and in the antisolar direction, in the sunspot. This is similar in the only other species which has been investigated, *Asterocampa leilia* (Rutowski 2000b). This way, they see a relatively dark part of the scene with low contrasts. We suggest that this dark and relatively even background will facilitate the detection of bright sunlit females passing through the sunspot. The larger the sunspot, the longer the passing female will reflect direct sunlight during its passage through the sunspot, increasing the chance of detection. Likewise, it will allow the perching male to spot an intruding males more easily. We In a study on *Asterocampa leilia* (Bergman et al 2015), a species that perches in the open, detection of females was found to be the best with a blue sky background. Assuming that butterflies, like bees, use green receptor contrast for achromatic contrast detection tasks, the blue sky will be similarly dark as the dark parts of the foliage background that perching *P. aegeria* males face.

Although we see a clear pattern, both in the way that males position themselves in a sunspot, and in the images seen from the perches, this analysis does not reveal how males choose a perching position in the first place. Studies by Bergman and Wiklund (2009) confirm that males prefer larger to smaller sunspots, but not how they choose the sunspot or the perch within the sunspot. Together with our description of the environmental light field experienced by males in sunspots, this seems to suggest that male *P. aegeria* have to fly around, visit sunspots and experience the light distribution and visual scene before choosing a perch. Indeed, males can be seen flying around and landing in different sunspots, returning more consistently to the larger and thus more attractive sunspots (M.B., personal observations). A closer analysis of this initial choice behaviour of perching male butterflies would be highly interesting. Moreover, the role of temperature deserves attention again, even though we did not find any clear pattern; for instance, unlike in *Asterocampa*, wing posture (open or closed) did not depend on sun elevation (Fig. 3).

### Visual acuity of male *P. aegeria* and detection distance for females

**Fig. 9:**
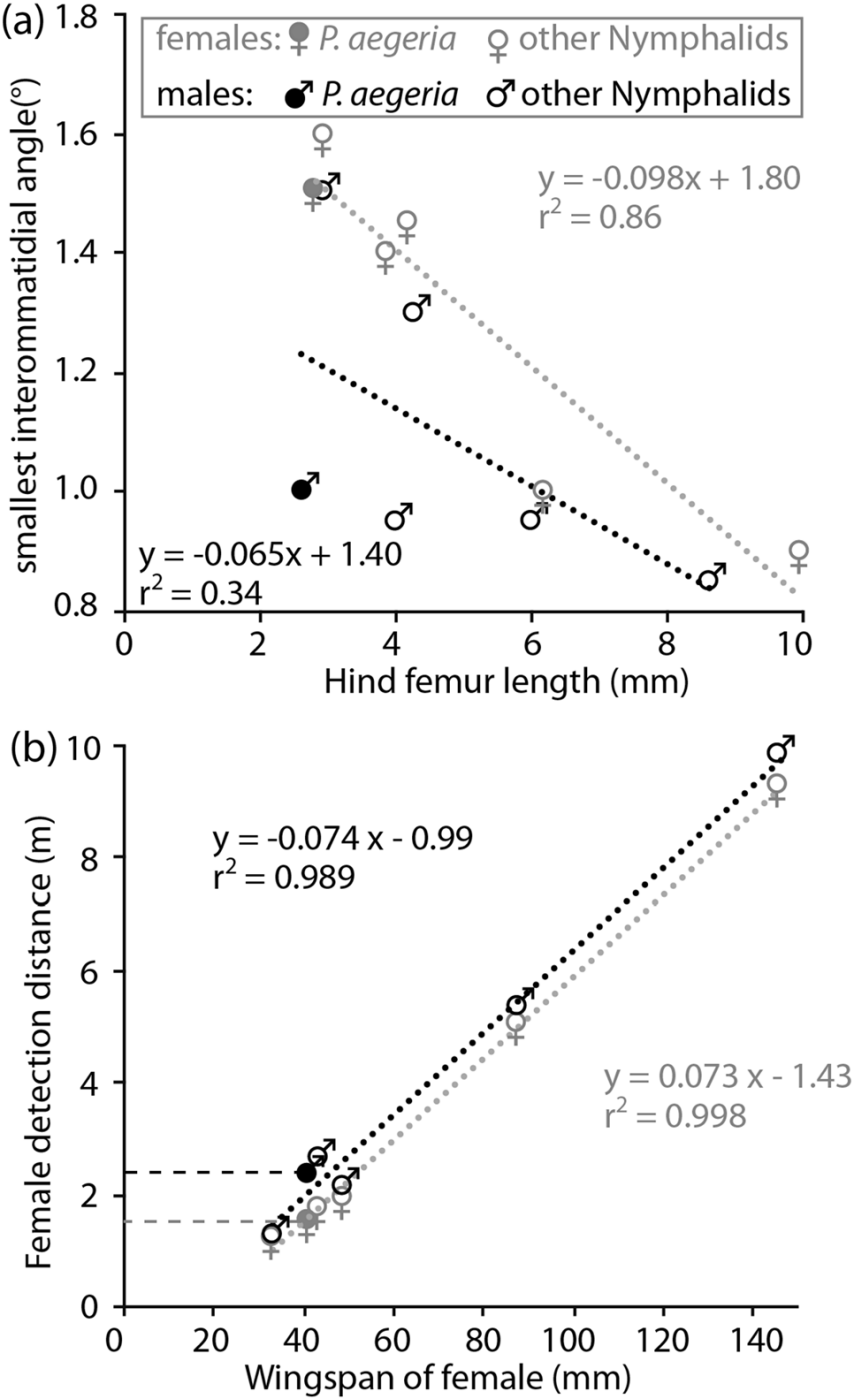
**a** Smallest interommatidial angles as a function of hind femur length (HFL) and **b** the distance at which a conspecific female is detected [using the wing span = double forewing length (FWL) as size estimate], as predicted from the smallest interommatidial angles (IA) in the male and female eyes, for six species of Nymphalids. *Pararge aegeria* (IA this study, HFL Rutowski 2000a, b, FWL Windig and Nylin 1999), other species, in increasing size: *Araschnia levana*, *Polygonium c-album*, *Asterocampa leilia*, *Parthenos sylvia*, *Caligo eurolochus* (data from Rutowski et al. 2009 and Rutowski and Warrant 2002, FWL and HFL of *A. leilia* Rutowksi 2017, unpublished data)

Eye size in butterflies is correlated with body size (Rutowski 2000a). Similar to *Asterocampa leilia* (Rutowski and Warrant 2002), but unlike some other species of nymphalid butterflies (Rutowski et al. 2009), *Pararge aegeria* has a clear sexual dimorphism in the interommatidial angles of the eye. Interommatidial angles of female nymphalids scale inversely with body size, thus larger species have smaller interommatidial angles allowing for higher spatial resolution (grey symbols and regression line in Fig. 9a). The interommatidial angles in the frontal visual field of male *P. aegeria*, however, are similar (1°) to those found in the eyes of other, much larger male nymphalids (black symbols in Fig. 9a; Rutowski and Warrant 2002; Frederiksen and Warrant 2008; Rutowski et al. 2009).

Rutowski et al. (2009) demonstrated that larger butterflies can detect conspecifics from much larger distances than smaller ones, a result of the combination of the larger body size itself and the higher resolution of their eyes. Following their reasoning and assuming that the acceptance angles of the species matches the interommatidial angles in the frontal visual field, leading to optimal sampling, we conclude that a flapping female *P. aegeria* with a forewing of 20 mm length (Windig and Nylin 1999) covers the visual field of a frontal ommatidium of the male at the distance of about 2.3 m (Fig. 9b), to cover the visual field of an ommatidium in the lateral of peripheral visual field of the male, the female has to come as close as 1.2–1.5 m. By comparison, the resolution of the female eye only allows the detection only from 1.5 m distance. These detection distances are given as the single object detection criterion set by Land (1997a, b), which does not take contrast sensitivity into account. With a contrast sensitivity of 10, realistic for an insect (males of honeybees and carpenter bees can detect a female reducing the light flux in a single ommatidium by as little as 6–8%; Vallet and Coles 1993; Somanathan et al. 2017), a female should be detectable already at longer distance.

The distance, from which a male *P. aegeria* can detect a passing female has not been tested empirically yet. However, Bergman and Wiklund (2009) tested the response of perching *P. eageria* males to a butterfly model, passing at a height and speed natural for the species. They found that the shorter the distance between the perched male and the passing model, the more likely the male was to respond. The detection limit has, to our knowledge, only been tested in one species. Rutowski et al. (2001) showed that perching males of *A. leilia* were unable to detect any passing object of the size of a female, at a distance longer than 3 m. The conservative prediction (see Fig. 9b) for this species is 2.6 m, confirming that high contrast sensitivity indeed allows mate detection from a distance longer than predicted by spatial resolution, even in butterflies.

### Locations, posture and orientation of perching *P. aegeria* males

The general behaviour of males observed in this study was similar to that described in earlier studies (e.g. Wickman and Wiklund 1983; Bergman et al. 2007; Bergman and Wiklund 2009). Despite the large body of literature on the species, here we provide the first detailed description of the body posture of perching males of *P. aegeria*. We found that, similar to males of *A. leilia* (Rutowski 2000b), males of *P. aegeria* were facing into the direction opposite of the sun and facing at around 45° above the horizon with their eye equator, the area of the highest resolution. The pitch angle is inversely correlated to the sun elevation, such that the sun is seen in the dorsal part of visual field where resolution is low. This way, they create a rather dark and uniform background of forest foliage, in front of which a flying female likely creates a high contrast signal. Evidently, in a larger sunspot, the female will be sun-lit over a longer part of her flight path, thus making detection even more likely.

We have only imaged this background in the human-visible range, knowing that the majority of photoreceptors in nymphalid eyes have a sensitivity peak at 530 nm. Still, we appreciate that taking the ultraviolet part of the spectrum into consideration might incur minor changes in the image, as has recently been demonstrated for birds (Tedore and Nilsson 2019).

Our behavioural observations reveal a clear pattern in body posture and orientation, but also indicate that the males utilize a relatively small part of the sunspot. Over three consecutive landings the distance between the perches was on average less than a meter, even though the median length of the sunspots were 8 m and the median width 4 m. That males often return to a small delimited area of a seemingly large territory, is seen in other territorial species, e.g. *A. leilia* (Rutowski et al. 2001). These small-scale behavioural preferences might also be driven by the visual environment requirements, given that some areas within a sunspot are more beneficial for visual detection of females than others. However, how the visual environment varies within a large sunspot and how this affects male behaviour within the territory can only be revealed by further studies.

## Conclusions: How do butterfly males find perching locations?

Using a new combination of methods, we have provided a first detailed description of male body and head posture, viewing direction, visual field and spatial resolution, as well as the visual environment for one of the most well-studied perching butterfly species, *Pararge aegeria*. We see that males position themselves in such a way that they create a background in front of which they likely have the best chance to detect a passing female. This builds a basis for future studies describing and analysing how males find and evaluate the optimal sunspot, and in the sunspot, the optimal perch. It also opens for the next major step in investigating male–female interactions, by recording female flight paths and projecting them into the visual field of perching males. Thus, the results strongly indicate that the behaviour of male *P. aegeria* can only be understood in the light of their visual ecology.

## Supplementary Information

Below is the link to the electronic supplementary material.

Supplementary file1 (DOCX 528 KB)

Supplementary file2 (XLSX 15 KB)

## Declarations

### Availability of data and materials

All data material will be made available by the authors on request.

## References

[CR1] Alcock J (1987). Leks and hilltopping in insects. J Nat Hist.

[CR2] Baker RR (1972). Territorial behaviour of the nymphalid butterflies, *Aglais urticae* (L.) and *Inachis io* (L.). J Anim Ecol.

[CR3] Bergman M, Wiklund C (2009). Visual mate detection and mate flight pursuit in relation to sunspot size in a woodland territorial butterfly. Anim Behav.

[CR4] Bergman M, Gotthard K, Berger D, Olofsson M, Kemp DJ, Wiklund C (2007). Mating success of resident versus non-resident males in a territorial butterfly. Proc R Soc B.

[CR5] Bergman M, Lessios N, Seymoure BM, Rutowski RL (2015). Mate detection in a territorial butterfly—the effect of background and luminance contrast. Behav Ecol.

[CR6] Collett TS, Land MF (1975). Visual control of flight behaviour in the hoverfly *Syritta pipiens* L.. J Comp Physiol.

[CR7] Cordero CR, Soberon J (1990). Non-resource based territoriality in males of the butterfly *Xamia xami* (Lepidoptera: Lycaenidae). J Insect Behav.

[CR8] Courtney SP, Parker GA (1985). Mating behaviour of the tiger blue butterfly (*Tarucus theophrastus*): competitive mate-searching when not all females are captured. Behav Ecol Sociobiol.

[CR9] Davies NB (1978). Territorial defence in the speckled wood butterfly (*Pararge aegeria*): the resident always wins. Anim Behav.

[CR10] Endler JA (1993). The color of light in forests and its implications. Ecol Monogr.

[CR11] Fischer K, Fiedler K (2001). Resource-based territoriality in the butterfly *Lycaena hippothoe* and environmentally induced behavioural shifts. Anim Behav.

[CR12] Frederiksen R, Warrant EJ (2008). Visual sensitivity in the crepuscular owl butterfly *Caligo memnon* and the diurnal blue morpho *Morpho peleides*: a clue to explain the evolution of nocturnal apposition eyes?. J Exp Biol.

[CR13] Kelber A, Jonsson F, Wallén R, Warrant E, Kornfeldt BE (2011). Hornets can fly at night without obvious adaptations of eyes and ocelli. PLoS ONE.

[CR14] Land MF (1997). The resolution of insect compound eyes. Israel J Plant Sci.

[CR15] Land M (1997). Visual acuity in insects. Ann Rev Entomol.

[CR16] Land MF, Eckert H (1985). Maps of the acute zones of fly eyes. J Comp Physiol A.

[CR17] Lederhouse RC (1982). Territorial defense and lek behavior of the black swallowtail butterfly, *Papilio polyxenes*. Behav Ecol Sociobiol.

[CR18] Lederhouse RC, Codella SG, Grossmueller DW, Maccarone AD (1992). Host plant-based territoriality in the white peacock butterfly, *Anartia jatrophae* (Lepidoptera, Nymphalidae). J Insect Behav.

[CR19] Menzel JG, Wunderer H, Stavenga DG (1991). Functional morphology of the divided compound eye of the honeybee drone (*Apis mellifera*). Tissue Cell Res.

[CR20] Nilsson D-E, Smolka J (2021). Quantifying biologically essential aspects of environmental light. J R Soc Interface.

[CR21] Parker G (1978). Evolution of competitive mate searching. Ann Rev Entomol.

[CR22] Paul R, Steiner A, Gemperlein R (1986). Spectral sensitivity of *Calliphora erythrocephala* and other insect species studied with Fourier interferometric stimulation (FIS). J Comp Physiol A.

[CR23] Rosenberg RH, Enquist M (1991). Contest behaviour in Weidemeyer’s admiral butterfly *Limenitis weidemeyerii* (Nymphalidae): the effect of size and residency. Anim Behav.

[CR24] Rutowski RL (1984). Sexual selection and the evolution of butterfly mating behavior. J Res Lepid.

[CR25] Rutowski RL (1991). The evolution of male mate-locating strategies in butterflies. Am Nat.

[CR26] Rutowski RL (2000). Eye size variation in butterflies: intra- and interspecific patterns. J Zool.

[CR27] Rutowski RL (2000). Postural changes accompany perch location changes in male butterflies *Asterocampa leilia* engaged in visual mate searching. Ethol.

[CR28] Rutowski RL, Warrant EJ (2002). Visual field structure in Empress Leilia, *Asterocampa leilia* (Lepidoptera, Nymphalidae): Dimensions and regional variation in acuity. J Physiol A.

[CR29] Rutowski RL, McCoy L, Demlong MJ (2001). Visual mate detection in a territorial male butterfly (*Asterocampa leilia*): effects of distance and perch location. Behaviour.

[CR30] Rutowski RL, Gislen L, Warrant EJ (2009). Visual acuity and sensitivity increase allometrically with body size in butterflies. Arthropod Struct Dev.

[CR31] Scott JA (1974). Mate-locating behavior of butterflies. Am Midl Nat.

[CR32] Shields O (1967). Hilltopping. J Res Lepid.

[CR33] Somanathan H, Warrant EJ, Borges R, Kelber A (2017). Visual adaptations for mate detection in the male carpenter bee *Xylocopa tenuiscapa*. PLoS ONE.

[CR34] Srinivasan MV, Lehrer M (1988). Spatial acuity of honeybee vision and its spectral properties. J Comp Physiol A.

[CR35] Suzuki Y (1976). So-called territorial behaviour of the small copper, *Lycaena phlaeas daimio* Seitz (Lepidoptera, Lycaenidae). Kontyu.

[CR36] Tedore C, Nilsson D-E (2019). Avian UV vision enhances leaf surface contrasts in forest environments. Nat Commun.

[CR37] Thornhill R, Alcock J (1983). The evolution of insect mating systems.

[CR38] Vallet AM, Coles JA (1993). The perception of small objects by the drone honeybee. J Comp Physiol A.

[CR39] Van de Velde L, Turlure C, van Dyck H (2011). Body temperature and territory selection by males of the speckled wood butterfly (*Pararge aegeria*): what makes a forest sunlit patch a rendezvous site?. Ecol Entomol.

[CR40] van der Kooi CJ, Stavenga DG, Arikawa K, Belusic G, Kelber A (2021). Evolution of insect colour vision: from spectral sensitivity to visual ecology. Ann Rev Entomol.

[CR41] Wickman PO (1985). Territorial defence and mating success in males of the small heath butterfly, *Coenonympha pamphilus* L. (Lepidoptera: Satyridae). Anim Behav.

[CR42] Wickman PO, Wiklund C (1983). Territorial defence and its seasonal decline in the speckled wood butterfly (*Pararge aegeria*). Anim Behav.

[CR43] Wiklund C, Boggs CL, Watt BW, Ehrlich PR (2003). Sexual selection and the evolution of butterfly mating systems. Butterflies—ecology and evolution taking flight.

[CR44] Wiklund C, Friberg M (2011). Seasonal development and variation in abundance among four annual flight periods in a butterfly: a 20-year study of the speckled wood (*Pararge aegeria*). Biol J Linn Soc.

[CR45] Windig JJ, Nylin S (1999). Adaptive wing asymmetry in males of the speckled wood butterfly (*Pararge aegeria*)?. Proc R Soc Lond B.

[CR46] Zeil J (1983). Sexual dimorphism in the visual system of flies: the compound eyes and neural superposition in bibionidae (Diptera). J Comp Physiol.

