# Supplementary material for: Seeing the world through the eyes of a butterfly: visual ecology of the territorial males of Pararge aegeria (Lepidoptera: Nymphalidae)
Source: J Comp Physiol A Neuroethol Sens Neural Behav Physiol. 2021 Oct 28;207(6):701–13. doi: 10.1007/s00359-021-01520-3 (PMC8568875; doi:10.1007/s00359-021-01520-3)
Supplement: Supplementary file 1 — Supplementary file1 (DOCX 528 KB) [file 359_2021_1520_MOESM1_ESM.docx]

**Supplementary figures for**

**Seeing the world through the eyes of a butterfly:**

**Visual ecology of the territorial males of *Pararge aegeria* (Lepidoptera: Nymphalidae)**

Martin Bergman, Jochen Smolka, Dan-Eric Nilsson & Almut Kelber


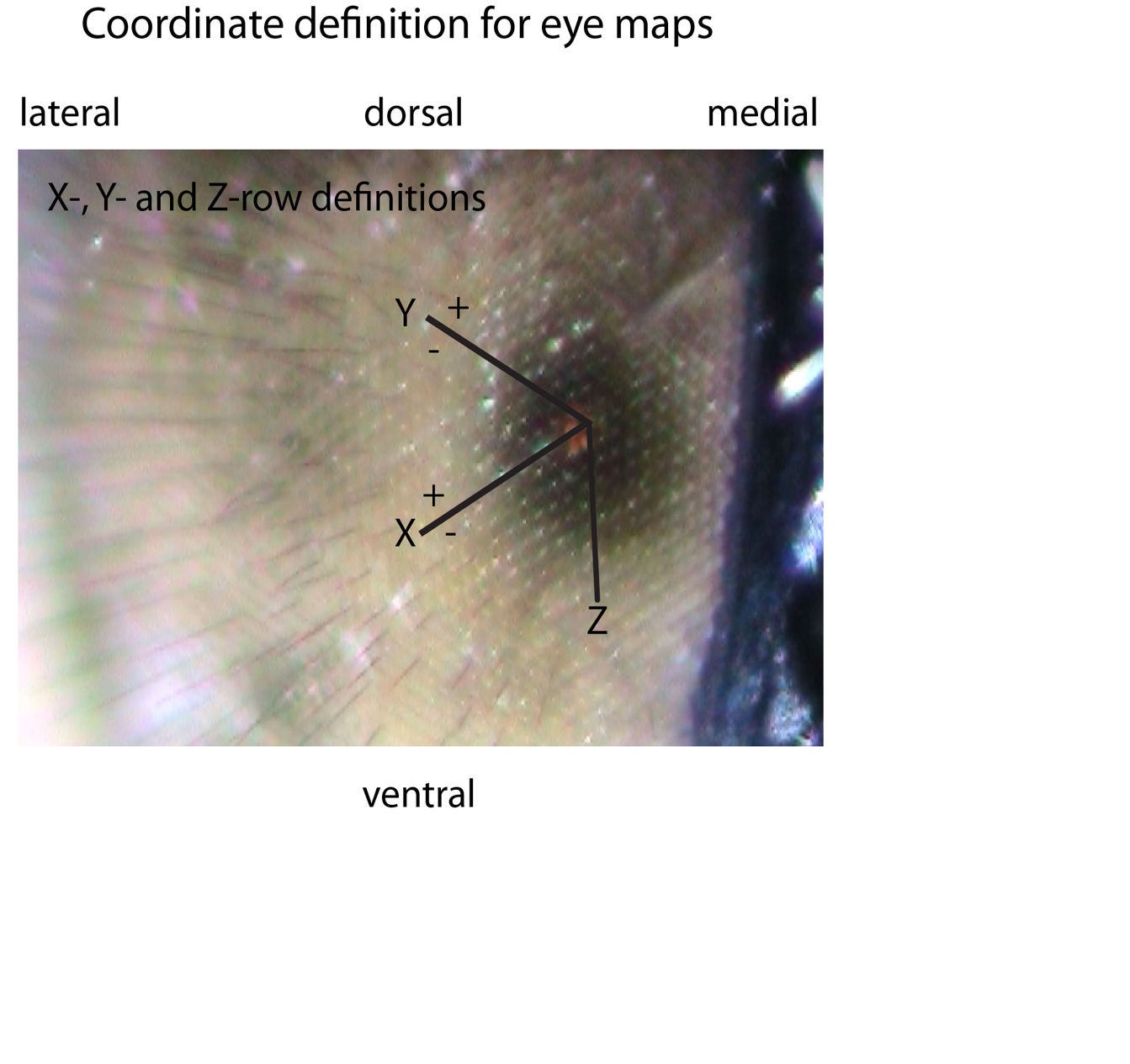


Figure S1. Coordinate definition for the eye maps. The x-, y- and z axes follow the hexagonal ommatidial mosaic. The position of the pseudopupil (facets with red eye glow elicited by the illuminating light) in each photo are given in x- and y-coordinates on the eye.


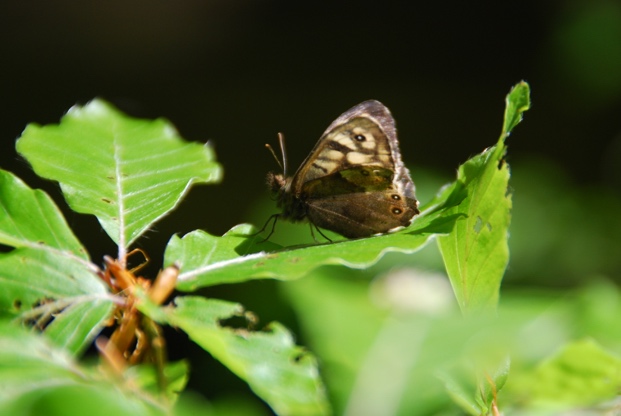

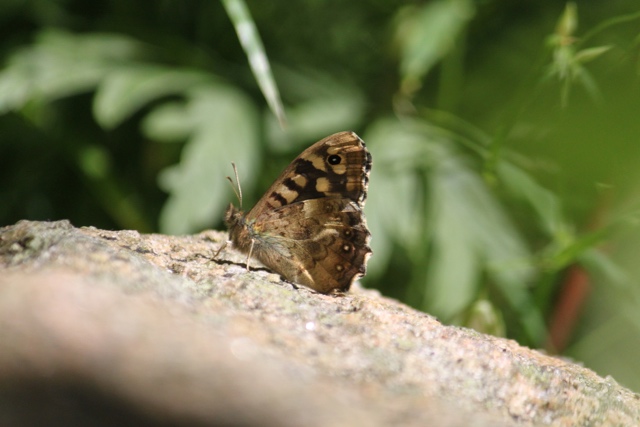


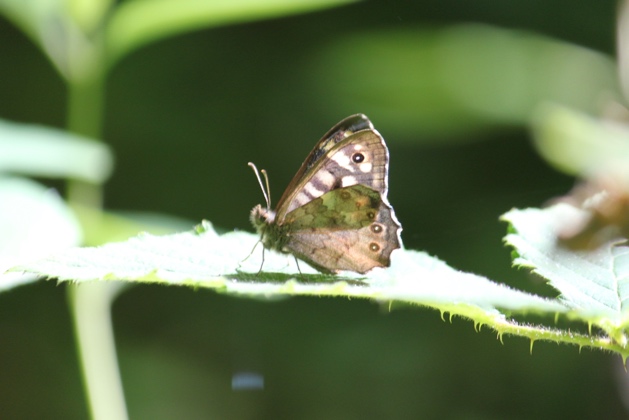

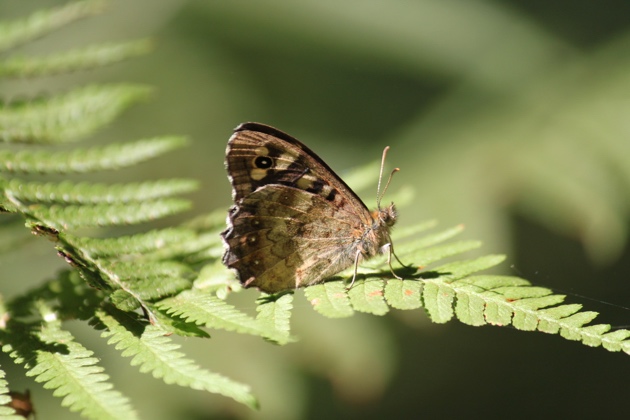


Figure S2. Examples of the photos of perching *P. aegeria* males that were used to determine body and head pitch.
